# Contact-Allergic Reactions to Cosmetics

**DOI:** 10.1155/2011/467071

**Published:** 2011-02-21

**Authors:** An Goossens

**Affiliations:** Department of Dermatology, Contact Allergy Unit, K. U. Leuven, 3000 Leuven, Belgium

## Abstract

Contact-allergic reactions to cosmetics may be delayed-type reactions such as allergic and photo-allergic contact dermatitis, and more exceptionally also immediate-type reactions, that is, contact urticaria. Fragrances and preservative agents are the most important contact allergens, but reactions also occur to category-specific products such as hair dyes and other hair-care products, nail cosmetics, sunscreens, as well as to antioxidants, vehicles, emulsifiers, and, in fact, any possible cosmetic ingredient. Patch and prick testing to detect the respective culprits remains the golden standard for diagnosis, although additional tests might be useful as well. Once the specific allergens are identified, the patients should be informed of which products can be safely used in the future.

## 1. Introduction

Nowadays almost everyone is using cosmetics products, which includes cleansing products such as soaps, bath and shower products, shampoos and toothpaste, as well as, for example, deodorants and make-up products. Indeed, they are used to clean, perfume, change the appearance, protect from body odours, and protect and keep the skin, teeth, and mucosal membranes in good condition. They differ from drugs because they lack diagnostic and therapeutic properties. 

Allergic reactions to cosmetic products are increasingly observed. The cosmetic allergens involved can reach the skin in several different ways: by direct application, by occasional contact with an allergen-contaminated surface, by airborne contact (e.g., vapours or droplets), by transfer by the hands to more sensitive areas (e.g., the eyelids), by a product used by the partner (or any other person), or be photo-induced, resulting from contact with a photo-allergen and exposure to sunlight, particularly UV-A light. An allergic contact dermatitis may sometimes spread (symmetrically) to other areas of the body not in direct contact with the allergen (id-like spread reaction); this is comparable to a reaction by systemic exposure (in which the allergen may reach the skin through the circulatory system and produce a systemic contact-type dermatitis), the latter being extremely rarely observed with cosmetics. Besides contact dermatitis, being a delayed allergic response, also immediate type reactions, that is, contact urticaria (syndrome), may exceptionally occur.

## 2. Allergic and Photoallergic Contact Dermatitis

### 2.1. Diagnosis

Physical examination and history taking frequently suggest the etiological factor(s), but patch (or epicutaneous) testing is used for diagnosing, with at least two readings of the test results, that is, at day 2 and 4/5 following their application. Allergen identification for a patient with a possible contact allergy to cosmetics is performed by means of patch testing with the baseline (standard) series, specific cosmetic-test series, the products used, along with their ingredients. With regard to the diagnosis of photoallergic contact dermatitis, photo-patch tests need to be performed: the allergens are tested in duplicate on the back and irradiated with U.V. light (most often UV-A 5 J/cm^−2^). Readings should be recorded immediately and 2 days, as well as at 3 or 4 days postirradiation.

Sometimes open or semiopen (or semiocclusive) tests [1, 2] and use tests [2, 3] or repeated open application tests (ROAT's) [4] are additional testing procedures. The former methods are useful modifications for cosmetics that have a slight irritation potential, such as, for example, cleansing products. With an open test, the substance is applied uncovered on the upper arm or upper back twice a day during at least 2 days (without washing the test site). A semiocclusive test consists of one application, with a cotton swab on about 1 cm^2^ of the upper back, of a minute amount of a test material, which is left to dry completely, and covered with acrylate paper tape. The readings are performed after 2, 3, and/or 4 days. Also diluted products (e.g., 1%-2% aqueous) can be tested this way. When contact allergy is suspected and patch testing with the product as such remains negative, use tests on the previously affected site or better, a repeated open application test (ROAT) can be performed. 

With ROAT, a very small amount or about 0.1 mL of test material is applied twice daily to the flexor aspect of the forearm near the cubital fossa, to an area approximately 5  ×  5 cm. The results are read after 1 week, but sometimes ROAT need to be performed up to 21 days, especially with low-concentrated allergens, to reveal an allergic reaction.

Once an allergen has been identified, it is the dermatologist's task to provide specific advice about the products that can be safely used, since subjects sensitive to specific ingredients must avoid products containing them. Although cosmetic labelling exists, providing the allergic patient with a limited list of cosmetics that can be used is, in our experience, most practical and effective [5].

### 2.2. The Allergens

In cosmetics, fragrances and preservative agents are the most important culprits, but reactions also occur to category-specific products such as hair dyes and other hair-care products, nail cosmetics, sunscreens, as well as to antioxidants, vehicles, emulsifiers, and, in fact, any possible cosmetic ingredient [6].

#### 2.2.1. Fragrance Components

They are frequent culprits in cosmetic allergies. Sensitization is most often induced by highly perfumed products, such as toilet waters, after-shave lotions, and deodorants, but fragrance-containing skin-care products may also cause reactions [6]. The main skin sites affected are the face, neck, axillae, and hands.

The literature confirms that the fragrance mix, which contains 8 perfume components (amyl cinnamal, cinnamal, cinnamyl alcohol, hydroxycitronellal, eugenol, isoeugenol, geraniol, and Evernia prunastri (oakmoss) extract, all diluted 1% in petrolatum and emulsified with sorbitan sesquioleate) and which is tested routinely in the baseline series, remains the best screening agent for contact allergy to perfumes because it can detect some 70% to 80% of all perfume allergies [7]. However, the literature also insists on the need to test with additional perfume allergens. Indeed, testing with additional markers such as, for example, other individual components, as well as with complex natural mixtures [8] increases the sensitivity of the testing. A new fragrance mix (II) consisting of hydroxyisohexyl 3-cyclohexene carboxaldehyde (Lyral), farnesol, citral, citronellol, coumarin, and alfa-hexyl cinnamaldehyde, as well as hydroxyisohexyl 3-cyclohexene carboxaldehyde [9] itself has been added to the baseline series as well [10]. Because of the increasing importance of fragrance allergy and to ensure that sensitized consumers are adequately informed, 26 fragrance components are (since March 2005) to be labeled as cosmetic ingredients on the packaging (Annex 3 of the Cosmetic Directive 2003/15/EC).

Multiple positive patch-test reactions are frequently associated with fragrance allergy and often indicate the presence of common or cross-reacting ingredients in natural products (e.g., also to plants of the *Compositae* or *Asteraceae *family) [11], the occurrence of cross-reactions between simple fragrance chemicals, or a concomitant sensitivity.

Fragrance components may be allergenic by themselves, but may also contain sensitizing oxidation products, as is the case with, for example, limonene [12] and linalool [13], and even certain contaminants. For example, resin acids and their oxidation products are the main allergens in colophony and are in Evernia furfuracea (tree moss) (a widely used perfume ingredient), as well as in oak moss, both in the qualities used by the industry, and in patch-test materials. Besides, in oak moss, atranol and chloratranol have been identified as the most potent allergens ever described [14].

#### 2.2.2. Preservatives

They are very imporant cosmetic allergens in water-based products such as cleansers, skin-care products and make-up [6]. However, within this class important shifts have occurred over the years [15].

Particularly methyldibromoglutaronitrile—that was used in a mixture with phenoxy-ethanol, better known as Euxyl K400—became such an important cosmetic allergen [6, 15] that the EU did not longer permit its further use in cosmetic products (March 2007). The methyl- and methylchloro-isothiazolinone mixture was commonly used in the 1980s and became a frequent cause of contact allergies, and is actually rising again. The mixture had been recommended (up to 15 ppm) to be used only in rinse-off products; however, it is still found in several leave-on products on the market such as moisturizers, and also in (baby) toilet tissue paper (wipes), which produces allergic contact dermatitis [16], most often in those who take care of the babies! Currently, the mixture is now being replaced by methylisothiazolinone alone, which is less allergenic but also less efficient as a preservative, hence larger use concentrations (up to 100 ppm) are required. Patients sensitized by the mixture often react to the nonhalogenated derivative as well though [17], and methylisothiazolinone is a primary sensitizer of itself. We recently observed several cases, most of which caused by the use of wipes (moist toilet paper) for intimate hygiene (and baby wipes); however, also other cosmetic products may be the sensitization source [18]. Since these preservative agents are widely used in household and industrial products, subsequent reactions frequently occur by contact (also airborne) with detergents, paints, glues, and so forth.

The incidence of positive reactions to formaldehyde and its releasers is slightly increasing again (data from the European Environmental Contact Dermatitis Research Group 2008, not published). Meanwhile, parabens are rare causes of cosmetic dermatitis and when allergy does occur, the sensitization source is most often a topical pharmaceutical product. This is often the case also for, for example, mefenesin, a rubefacient in topical pharmaceutical products, which cross-reacts with chlorphenesin, used as a preservative agent in cosmetics (data on file) and thus a potential sensitizing agent [19]. A further recently introduced preservative is iodopropynyl butylcarbamate, also present in baby and make-up cleansing wipes [20], first reported as a cosmetic allergen by Pazzaglia and Tosti in 1999 [21]. Its presence in cosmetics is being discussed, not because of its potentially allergenic properties, but because of its iodine content.

#### 2.2.3. Antioxidants

They are only a minor group of cosmetic allergens. Examples are propyl gallate, octyl gallate [22], which may cross-react with other gallates and are also used as food additives, and t-butyl hydroquinone, a well-known allergen in the United Kingdom but not in continental Europe. Some antioxidants are used more specifically in sunscreen products and also in moisturizing products to prevent ageing, but are rare causes of allergic contact dermatitis in such preparations, for example, tocoferol (vitamine E) acetate and retinol palmitate [23], and ascorbic acid (vitamin C) [24], and more recently also idebenone or hydroxydecyl ubiquinone (a synthetic analog of Coenzyme Q10 (CoQ10) [25]. Also sulfite-allergy may explain reactions to certain cosmetic creams, cleansing products, as well as hair dyes.

With regard to *category-specific ingredients*, the number of reactions to oxidative type *hair dyes* (PPD and related compounds) increases in some centers and do decrease in others [26]. Active sensitization to PPD and related compounds from temporary tattoos has become an epidemic, for example, [27]. Moreover, also immediate-type reactions or the contact-urticaria syndrome may occur [28] (see also below). Hair dyes are important causes of dermatitis both in clients, in whom they often cause severe reactions [29] and hairdressers. Other allergens in hairdressing products [26] are bleaches (persulfates, also causes of immediate-type allergic reactions), permanent-wave solutions (primarily glyceryl mono-thioglycolate which may provoke cross-sensitivity to ammonium-thioglycolate), and sometimes shampoo ingredients, for example, cocamidopropyl betaine and preservative agents. 

In *nail cosmetics*, tosylamide/formaldehyde (= toluenesulfonamide formal-dehyde) resin is the classical allergen and is the cause of “ectopic” dermatitis due to nail lacquer, which also may contain epoxy and (meth) acrylate compounds, as well as certain allergenic copolymers; it often gives rise to confusing clinical pictures, even mimicking occupational dermatitis. (Meth) acrylates are important causes of reactions to artificial nails preparations, more recently to gel formulations being the newest development in this regard, both in clients but particularly in manicurists [30].

Because of media attention being given to the carcinogenic and accelerated skin-aging effects of sunlight, *sunscreens* are increasingly used, not only in sunscreen products but also in other cosmetic products, including moisturizers. They may be responsible for allergic and photoallergic reactions [31] and, for example benzophenones sometimes also for contact urticaria (see below). In the past, dibenzoylmethane derivatives have been recognized as being important allergens [6, 32] and isopropyl dibenzoylmethane was withdrawn from use in cosmetics for this reason. On the other hand, methylbenzylidene camphor, cinnamates, and phenylbenzimidazole sulfonic acid are only occasional, sometimes even rare, causes of cosmetic reactions. Octyl triazone [33] rarely causes problems, but octocrylene [34] is increasingly reported as a contact allergen, often in children, and also frequently causes photo-contactallergic reactions [35], and, as with benzophenones, particularly in relation to photocontact sensitization by ketoprofen, a nonsteroidal anti-inflammatory drug used to treat muscle pain. This is most probably based on cross-reactivity.

The contribution of sunscreens to cosmetic allergy has been considered to be relatively small despite the increase in their use; however, the low rate of allergic reactions observed may well be because a contact allergy or a photo-allergy to sunscreen products is often not recognized, since a differential diagnosis with a primary sun intolerance is not always obvious. Furthermore, the patch-test concentrations generally used might be too low, in part because of the risk of irritancy. Last but not least, photo-patchtesting is not at all routinely performed in a dermatologic practice!

#### 2.2.4. Excipients, Emulsifiers, Surfactants, and Humectants

They are common ingredients to topical pharmaceutical and cosmetic products. The classical contact-allergens reported were rare cosmetic allergens, such as wool alcohols, fatty alcohols (e.g., cetyl alcohol), and propylene glycol [6], but more recently introduced ingredients are also described: for example, dicaprylyl maleate [36], isononyl isononanoate and trioley phosphate [37], and the humectants butylene glycol [38] and pentylene glycol [39], being aliphatic alcohols with similar uses (solvent, humectant, and antibacterial) to propylene glycol that is considered to be more irritant and allergenic. Surfactants, in particular, have long been regarded as irritants, but their sensitization capacities should not be overlooked. It is imperative, of course, that patch testing be properly performed to avoid irritancy and that the relevance of the positive reactions be determined. Some of these substances are also, because of their low irritancy potential and “skin-mildness”, often incorporated in skin-care products “recommended by dermatologists”, “for use on intolerant” or “for sensitive skin” that have become very popular in recent years. A low irritant potential, however, does not preclude the occurrence of, albeit rarely, allergic contact dermatitis from such cosmetics. Examples are cocamidopropylbetaine that is considered to be more irritant than allergenic though, and alkyl glucosides, that is, condensation products of fatty alcohols with glucose such as coco-and lauryl glucosides [40], which are often used as mild surfactants and cleansing agents, as well as emulsifiers, particularly cetearyl- and decyl glucoside, the latter exceptionally being a hidden allergen in sunscreens [41].

Other possible allergens include ethylhexylglycerin (syn.: octoxyglycerin), a skin conditioning agent [42], and methoxy PEG-17 and PEG-22/dodecyl glycol copolymers (alkoxylated alcohols and synthetic polymers used as emulsion stabilizers and suspending and viscosity-increasing agents, and also as skin-conditioning agents) [43, 44]. Moreover, also other copolymers present in nail lacquers, and skin-care and sunscreen products have been reported as skin sensitizers [45]. The exact sensitizing component in them, however, remains unknown.

#### 2.2.5. Natural Ingredients

Plant extracts and herbal remedies have become very popular in recent years and may give rise to (sometimes severe) contact dermatitis problems [46, 47]. Patients allergic to perfume components should be advised to avoid cosmetics containing plant extracts (separately labelled on the packaging with their Latin INCI-name), some of which being fragrance ingredients such as essential oils [48] and which are not recognized as such by the consumers, or that are used because of other properties, for example, antibacterial or antifungal, such as, for example, *Melaleuca alternifolia* or tea tree oil [49].

Protein-derived ingredients, in particular, are often used in skin-care products, especially in those for treating dry skin in atopic subjects (often children). Allergic contact dermatitis (sometimes located mainly on the eyelids) from, for example, oat meal (*Avena*) [50], hydrolysed wheat [51], or soyabean extracts [52] may develop occasionally, but such products more frequently induce immediate-type reactions (see below).

## 3. Contact Urticaria (Syndrome)

Contact urticaria appears immediately (mostly within 5 to 20 minutes, exceptionally later) upon contact with the causal agent. The skin reaction is clinically characterized by redness and oedema (sometimes urticarial papules), and may, when immunologically mediated, be accompanied by extracutaneous symptoms such as conjunctivitis, respiratory problems, dizziness, and even anaphylaxis. This is referred to as the “contact urticaria syndrome” in which 4 stadia can be recognized.

Cutaneous symptoms:

Stadium 1: localised urticaria,
Stadium 2: generalised urticaria, extracutaneous symptoms,
Stadium 3: bronchial asthma, rhinoconjunctivitis, otolaryngeal, gastrointestinal symptoms,
Stadium 4: anaphylaxis.

### 3.1. Diagnosis

The diagnosis of contact urticaria consists of a careful history, inspection of the clinical symptoms, and the performance of immediate tests: the suspected materials are tested as such (open), but mostly with prick testing. Readings are performed immediately and up to 1 hour. Also a provocation or usage test can be performed. However, if the anamnesis or the clinical symptoms observed point to a severe extracutaneous reaction, attention is to be paid not to elicit a severe reaction on testing, which should be performed in a hospital environment only. In case of an immunologic-mediated urticaria, specific IgE-antibodies can be searched for. Indeed, there also exists nonimmunologic contact urticaria (NICU), for example, caused by chemicals, such as cinnamal (a fragrance component), and sorbic and benzoic acids (preservatives).

### 3.2. The Allergens

Cosmetic examples of substances to which also severe reactions have been reported are permanent hair dyes containing PPD [28, 29, 53], the sunscreen agents benzophenones [54–56], and hair bleaches, that is, persulfates, which are often responsible for respiratory symptoms in hairdressers [57]. However, not only low-molecular chemicals but also proteins and protein-derived, that is, hydrolysed products used in skin- and hair-care products are increasingly reported in this regard: for example, oat or *Avena* extract [58, 59]—of which the allergenic proteins may be removed though [60]—hydrolyzed wheat protein [61]. Immediate-type reactions to protein-derived products may sometimes give rise to severe symptoms [61, 62]. Although such reactions seem to be rare and may sometimes be irritant in nature, especially in atopic subjects, their use has given rise to controversies since subjects may perhaps get sensitized through topical preparations and develop food allergies afterwards, or vice-versa, and also in children [58–60].

## 4. Conclusions

Fragrance components and preservatives are the most frequent cosmetic contact allergens; however, all ingredients must be considered as potential culprits and patchtested. Besides allergic contact dermatitis, also immediate-type reactions may occur, for which prick tests are the golden standard for diagnosis. Once the specific allergens are identified, the patient should be informed on which products can be safely used in the future. Indeed, the so-called “hypoallergenic” products are not necessarily less sensitizing [63].
